# Roles of NFκB-miR-29s-MMP-2 circuitry in experimental choroidal neovascularization

**DOI:** 10.1186/1742-2094-11-88

**Published:** 2014-05-15

**Authors:** Jingjing Cai, Guibin Yin, Bing Lin, Xianwei Wang, Xiaoling Liu, Xiaoyan Chen, Dongsheng Yan, Ge Shan, Jia Qu, Shengzhou Wu

**Affiliations:** 1 School of Optometry and Ophthalmology and Eye Hospital, Wenzhou Medical University, 270 Xueyuan Road, Wenzhou, Zhejiang 325003, People’s Republic of China; 2 State Key Laboratory Cultivation Base and Key Laboratory of Vision Science, Ministry of Health, China and Zhejiang Provincial Key Laboratory of Ophthalmology and Optometry, 270 Xueyuan Road, Wenzhou, Zhejiang 325003, People’s Republic of China; 3 School of Life Sciences & CAS Key Laboratory of Brain Function and Disease, University of Science and Technology of China, 443 Huangshan Road, Hefei, Anhui Province 230027, People’s Republic of China

**Keywords:** Choroidal neovascularization (CNV), Matrix metallopeptidase-2 (MMP-2), Nuclear factor kappa-light-chain-enhancer of activated B cells (NFκB), microRNA-29 family (miR-29s), Tumor necrosis factor alpha (TNFα)

## Abstract

**Background:**

Previous reports have indicated that matrix metallopeptidase-2 (MMP-2) regulates angiogenic processes, which are involved in choroidal neovascularization (CNV). However, the regulation of MMP-2 in CNV has not been well-characterized. To gain more information about the regulation of MMP-2 in CNV, we analyzed the circuitry associated with MMP-2 regulation in a CNV model and in cell cultures, focusing on NFκB and the microRNA-29 family (miR-29s).

**Methods:**

The CNV model was established by subjecting C57BL/6 mice to fundus photocoagulation with a krypton red laser. In choroidal-retinal pigment epithelial (RPE) tissues of the model, immunohistochemistry was used to evaluate the angiogenesis and MMP-2 expression; reverse-transcription quantitative PCR (RT-qPCR) was used to determine the levels of miR-29s; and western blot was used to analyze the protein levels of nuclear factor kappa-light-chain-enhancer of activated B cells (NFκB) inhibitor, IκBα, and its phosphorylated form, phospho-IκBα. At the cellular level, RT-qPCR was used to examine the levels of miR-29s following NFκB activation by tumor necrosis factor alpha (TNFα); and western blot and luciferase assay were used to determine the regulation of MMP-2 by miR-29s in a human RPE cell line (ARPE-19) and in an umbilical vein endothelial cell line (EA hy926).

**Results:**

MMP-2 staining was increased in the choroidal neovascular membrane of laser-treated retina. Also, the NFκB pathway was induced in choroid-RPE tissue, as evidenced by a lower protein level of IκBα and a higher level of phospho-IκBα in the tissue homogenates than in those from non-treated eyes. During the period when the NFκB pathway was induced, reduced miR-29s were detected in the choroidal-RPE tissue of the laser-treated eyes. In cultured ARPE-19 cells, TNFα decreased miR-29a, b, and c, and the effects were rescued by NFκB decoy. In ARPE-19 and EA hy926, miR-29s mimics reduced the contents of secreted MMP-2 in the culture media. We also documented that miR-29s reduced MMP-2 3’-UTR-mediated luciferase transcription.

**Conclusions:**

The results suggest that in CNV, NFκB activation inhibits miR-29s, which may contribute to angiogenesis by up-regulating the MMP-2 protein level in RPE cells. These observations may help in developing a strategy for resolving CNV by targeting miR-29s levels.

## Introduction

Choroidal neovascularization (CNV) occurs in wet-type age-related macular degeneration, extreme myopia, and pathological myopia [1-3]. Angiogenesis in CNV is a complicated process with imbalance in the production of pro-angiogenic and anti-angiogenic factors (for example, excessive production of vascular endothelial growth factor (VEGF)), leading to pathological neovascularization [4]. The process of angiogenesis as it occurs in CNV involves the proteolysis of an extracellular matrix, the proliferation and migration of endothelial cells, and the synthesis of a new matrix in the destination [5].

Matrix metalloproteinase (MMP) family proteins play important roles in angiogenesis partly due to their ability to degrade basal membrane and extracellular matrix proteins. MMPs are secreted as zymogens, which require cleavage to create active forms of the enzyme. For instance, matrix metalloproteinase-2 (MMP-2) is derived from pro-MMP-2 by MT1-MMP cleavage and MMP-9 is derived from pro-MMP9 by MMP3 cleavage [6]. The activity of MMP is also down-regulated by endogenous inhibitors, namely tissue inhibitors of metalloproteinase [7]. Recent studies indicate that MMP-2 is indispensable in forming CNV because reduced CNV or attenuated retinal angiogenesis are observed in MMP-2 deficient mice [8,9]. RPE is a major resource for producing MMP-2 in the retina and overexpression of VEGF and MMP-2 by RPE is critical for angiogenesis in CNV [10,11].

MicroRNAs (miRNAs) are a family of endogenous, non-coding small RNAs with mature-type sizes ranging from 19 to 25 nucleotides [12]. In eukaryotes, miRNAs bind to the complementary sequences of the targeted mRNA, mostly 3’-untranslated region (3’-UTR), leading to translation repression or sometimes mRNA degradation [12]. Approximately 50% of miRNAs constitute clusters in a chromosome and transcribe as polycistronic primary transcripts [13]. Specifically, the microRNA-29 family (miR-29s) consists of a miR-29a/b1 cluster in one chromosome and a miR-29b2/c cluster in a different chromosome. Recent data indicate that miR-29s have multiple functions via binding to the 3’UTR of the target mRNAs: regulation of methylation status in lung cancer via targeting DNA methyltransferases 3A and 3B [14]; suppression of tumor angiogenesis, invasion and metastasis by regulating MMP-2 expression [15]; regulation of the processing of the β-amyloid precursor protein by decreasing β-secretase expression [16]; and activation of p53 via suppressing p85 alpha and CDC42, which both negatively regulate p53 [17]. Regulation of miR-29s also occurs in different ways: NFκB-YY1 pathway activation regulates skeletal muscle differentiation via inhibition of miR-29s transcriptions [18], and miR-29s is up-regulated in a p53-dependent manner in circumstances of aging and chronic DNA damage [19].

Recent studies have suggested that NFκB plays important roles in the early stage of angiogenesis [20,21], in CNV [22]; the inhibition of miR-29s by NFκB occurs in various cells [18,23]; and MMP-2 clearly contributes to angiogenesis [5]. These lines of research prompted us to examine the relationships among the NFκB pathway, miR-29s, and MMP-2 *in vivo* and *ex vivo*, with focus on an angiogenesis model, in other words experimental CNV.

## Materials and methods

### Materials

The following substances, materials, and reagents (and suppliers) were used in this study: the miRCURY LNA™ Universal RT microRNA PCR system including universal cDNA synthesis kit, SYBR™ Green Master Mix, and the primers for has-miR-29s and 5 s RNA (Exiqon, Vedbaek, Denmark); Endofree Qiagen plasmid maxi kit (QIAGEN Gmbh, Hilden, Germany); Dual-Glo™ luciferase kit and FuGENE™ HD transfection reagent, pGEM-T vector (Promega Branch, Beijing, China); Ex Taq and restriction enzymes (Takara, Dalian, China); Lipofectamine™ 2000 transfection reagent, Trizol, DNaseI (Invitrogen, Shanghai, China); miR-29a, b and c mimics (Ambion, Austin, Texas, United States); pMIR-REPORT™ vector (Applied Biosystems, Foster City, California, United States); 3-amino-9 ethylcarbazole (AEC) and VECTASTAIN Elite ABC kit (Vector Laboratories, Burlingame, California, United States); MMP-2 antibodies (Cell Signaling Technology, Beverly, Massachusetts, United States; Lifespan Biosciences, Atlanta, Georgia, United States); CD31 antibody (BD PharMingen, San Diego, California, United States); ARPE-19 and HEK 293 cells (ATCC, Manassas, Virginia, United States); Dulbecco’s Modified Eagle Medium (DMEM), DMEM/F-12, Fetal Bovine Serum (FBS), Opti-MEM (Gibco, Gaithersburg, Maryland, United States); and Neg-50 frozen section media (Microm BioResource, Auburn, California, United States).

### Establishment of a CNV model

CNV was induced in C57BL/6 mice at the age of between 6 and 8 weeks, with 6 burns surrounding the optic nerve by krypton red laser photocoagulation (50 μm spot size, 0.05 second duration, 260 mW) as previously described [24,25]. The right eyes were treated with laser, and the left eyes were left untreated, serving as the control. A bubble formed at the laser spot, indicating rupture of the Bruch’s membrane. The animals were euthanized between days 12 and 15 in order to assess the angiogenesis, and at days 3 and 5 for examining protein levels of NFκB and miR-29s expression. The laser spots were evaluated for the presence of CNV using hematoxylin and eosin staining and immunohistochemistry against CD31.

### Tissue preparation

CNV was induced in mice as described above. The animals’ eyes were enucleated at various time points. For immunohistochemistry analysis, eyes were imbedded in Neg-50 frozen section media and immediately frozen in liquid nitrogen. The samples were kept at -80°C before cryostat sectioning. For western blot and reverse-transcription (RT-qPCR) analyses of choroid-RPE tissue, the anterior segments, the vitreous, and the neurosensory retina of the eyes were removed, and the choroid-RPE layer was scraped from the sclera.

### Immunohistochemistry analysis

The frozen eyeballs were cryosectioned at 10 μM thickness. After fixation in acetone for 12 minutes at 4°C and blocking with goat serum, the cryosections were incubated overnight with primary antibody (CD31, 1:20; MMP-2, 1:100). The sections were then washed with PBS and subsequently incubated with biotinylated secondary antibody for 30 minutes at room temperature, followed by incubating with avidin-horseradish peroxidase complex (VECTASTAIN Elite ABC kit) for 30 minutes at ambient temperature. AEC was used to detect the peroxidase activity, after which the sections were counterstained with hematoxylin.

### Cell culture, treatment and transfection

ARPE-19 cells were cultured in DMEM/F-12 supplemented to 10% with FBS. EA hy926 and HEK-293 cells were cultured in DMEM supplemented to 10% with FBS. The cultures were maintained in a humidified 5% CO_2_ incubator at 37°C. Cells were passaged by use of 0.05% trypsin/ Ethylenediaminetetraacetic acid (EDTA) (Life Technologies, Gaithersburg, United States). Before transfection, the culture media for ARPE-19 cells were switched to DMEM/F12 supplemented to 1% with FBS for 2 hours. Then, ARPE-19 cells were transfected with 50 nM NFκB decoy oligodeoxynucleotides (ODN) or scrambled ODN with FuGENE™ HD transfection reagent and incubated for another 4.5 hours at 37°C. The cultures were treated with tumor necrosis factor alpha (TNF-α)(10 ng/mL) for 12 hours at 37°C and harvested for RT-qPCR analysis.

### Western blot analysis

For detecting secreted MMP-2 contents from supernatants with immunoblotting, the conditioned media were collected and concentrated with an Eppendorf concentrator 5301 (Eppendorf AG, Hamburg, Germany). The loading volumes in SDS-PAGE gels from control and treated conditions were adjusted so that the loading from each condition originated from equal protein mass in culture dishes [26].

For MMP-2 immunoblotting, ARPE-19 and EA hy926 cells were seeded onto six-well plates and transfected with miR-29s mimics (50 nM) using Lipofectamine™ 2000 in Opti-MEM media, according to the manufacturer’s instructions. At 4 to 6 hours after transfection, the cells were switched to DMEM/F12 supplemented to 10% with FBS for another 48 hours and collected for analyses. The MMP-2 antibody (1:1000) and the peroxidase-conjugated secondary antibody (1:5000) were used, followed by enhanced chemiluminescence (ECL) detection.

For NFκB analysis, choroidal-RPE tissues were homogenized with protein lysis buffer containing protease inhibitor cocktail and centrifuged to remove insoluble material. The blotting procedures followed the protocol previously described [27].

### Reverse-transcription quantitative real-time PCR

Total RNAs from choroidal-RPE tissue and cultured ARPE-19 cells were isolated using Trizol reagent. To prevent genomic contamination, RNA was further purified with DNase I (300 ng RNA was treated with 1 μL DNase I at room temperature for 15 minutes and 65°C for 10 minutes after addition of 2.5 mM EDTA). RNA (60 ng) was used to synthesize the cDNA with a cDNA synthesis kit (Exiqon) with deoxy-thymine nucleotide oligomer (oligo-dT) as the primer. The cDNA product (4 ng) was used as a template for PCR reaction with locked nucleic acid (LNA™) primer sets (Exiqon) targeting has-miR-29s and 5 s was used as an internal reference gene. The miRCURY LNA™ SYBR Green was used as reaction dye, and all PCR reactions occurred in a 96-well ABI plate format in ViiA™ 7 real-time PCR system (Applied Biosystems). The relative quantification method (a delta-delta C(T)) was adopted and 40 cycles of PCR reactions (95°C for 10 seconds and 60°C for 1 minute) were started with 50°C for 2 minutes, followed by 95°C for 10 minutes. Dissociative curves were used to confirm the reaction specificity.

### Plasmid construction and luciferase reporter assay

The incorporation of 3’UTR of human MMP-2 into multiple cloning sites of pMIR-REPORT™ vector involved two steps of cloning strategy: first, ligation of the 3’UTR PCR product into pGEM-T vector, named T1-MMP2-3’-UTR, and second, introduction of new restriction sites on the PCR product, with T1-MMP-2-3’-UTR as the template. The PCR product was then subcloned into pMIR-REPORT™. Briefly, the PCR product of the 3’UTR of the human MMP-2 was amplified from the genomic DNA of ARPE-19 cells with the forward primer: 5’-TTC CTC TCC ACT GCC TTC GAT A-3’ and the reverse primer: 5’-AGG ACA GAG GGA CTA GAG CTT A-3’ corresponding to the sequences from 2319 to 2340 bp and from 3257 to 3278 bp respectively, in the human MMP2 mRNA 3’-UTR (GenBank, NM_004530.4). The PCR began at an initial cycle (94°C for 30 seconds, 62°C for 30 seconds, and 72°C for 45 seconds), followed by serial cycles with a Tm stepdown of 1°C per cycle until Tm = 52°C was reached, and the cycle was then repeated 25 times. A total of 35 reaction cycles were performed with Takara LA Taq (Takara), preceded by a denaturation of 4 minutes at 94°C and finished with a 10-minute extension at 72°C. After confirming the presence of PCR product with sequencing, the regular PCR method (instead of touchdown PCR) was adopted with the forward primer: CATG *ACT AGT* CCT CTC CAC TGC CTT CGA TA (SpeI restriction site is indicated italic) and the reverse primer: CATG *AAG CTT* AGG ACA GAG GGA CTA GAG CT (HindIII restriction site is indicated italic). A total of 30 reactions were conducted (94°C for 30 seconds, 52°C for 30 seconds, and 72°C for 45 seconds). The PCR reactions were concluded with a 10-minute extension at 72°C. The resultant vector was named as pMIR-MMP-2 3’UTR.

HEK-293 cells were transfected in serum-free DMEM into 24-well plates with 50 ng pMIR-MMP-2 3’UTR containing firefly luciferase coding sequence, 25 ng pRL-SV40 Renilla vector (Promega Branch, Beijing, China) and 5 nM miR-29 mimics or negative control mimics (NC). The plasmid mixtures were incubated with Lipofectamine™ 2000 in opti-MEM for 30 minutes, transfected to the HEK-293 cells for 3 hours, washed away with DMEM, and then switched to regular culture media, DMEM plus 10% FBS. At 24 hours after transfection, Firefly and Renilla luciferase activities were measured with a dual luciferase reporter assay kit (Promega Branch, Beijing, China) according to the manufacturer’s instructions.

### Decoy oligodeoxynucleotide (ODN)

Double-stranded ODNs used as NFκB decoy were synthesized as previously described [28]. The sequences were 5'-CCT TGA AGG GAT TTC CCT CC -3' and 3’-GGA GGG AAA TCC CTT CAA GG-5’. Scramble ODNs served as control decoy, and the sequences were 5’-TTG CCG TAC CTG ACT TAG CC-3’ and 3’-AAC GGC ATG GAC TGA ATC GG-5’.

### Statistical analysis

Data were analyzed for significant difference (*P* < 0.05) with a one way analysis of variance (ANOVA) and Bonferroni *post hoc* test for multiple comparisons (SPSS 15.0.1; SPSS Inc., Chicago, Illinois, United States).

## Results

### Increased CNV and MMP-2 staining in the choroid-RPE membrane of the CNV model

**Figure 1:**
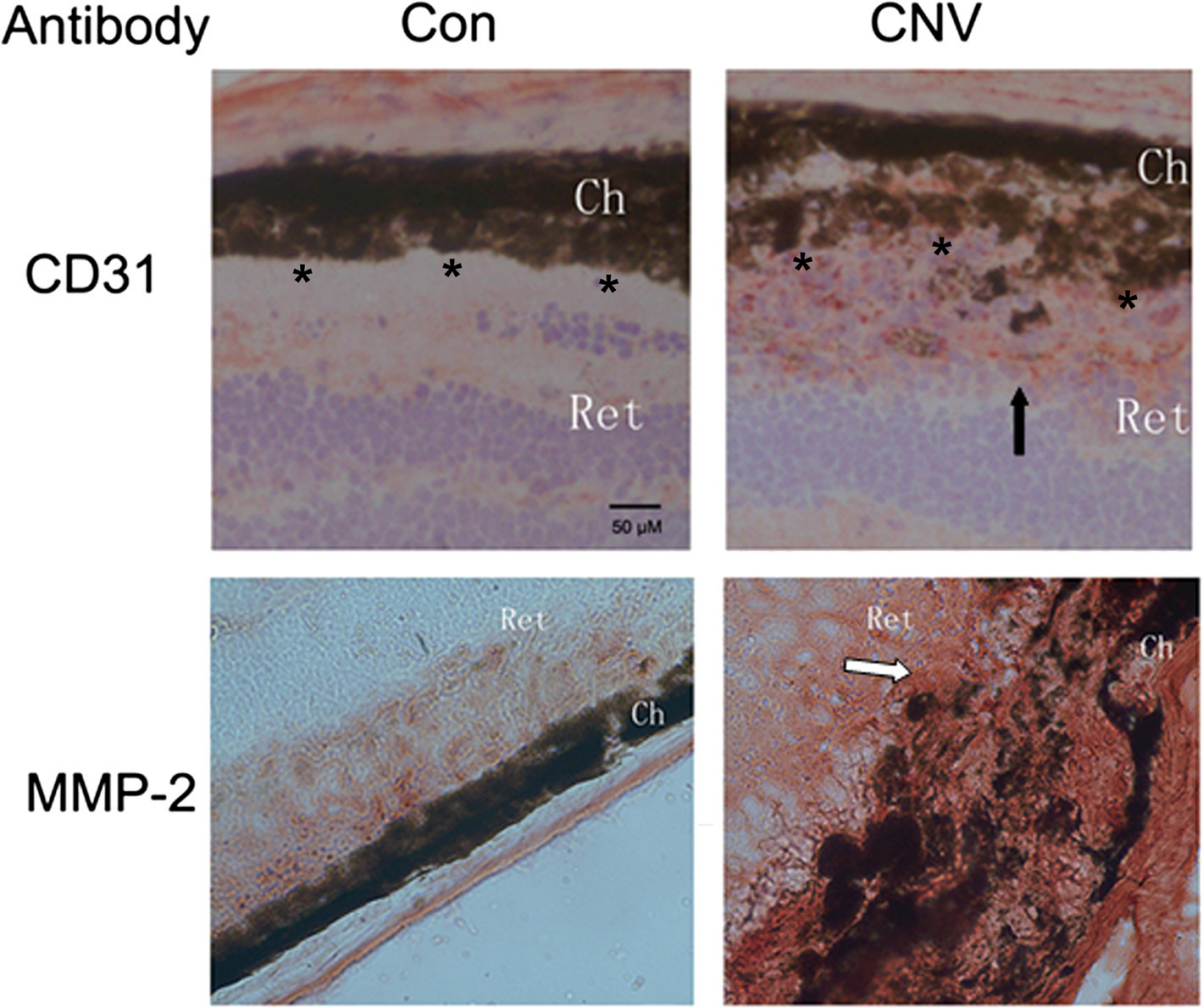
**Increased MMP-2 staining in the choroidal-RPE membrane of CNV model.** Cryosections from non-treated (left panel) and laser-lesion (right panel) eyeballs between 12and 15 days after photocoagulation were processed for CD31 (top row, dark arrow) and MMP-2 staining (bottom row, white arrow) as described in Methods. *marks the margin of the retinal pigment epithelial cell layer (top row). The images (from control or CNV) are typical of those in triplicate experiments and the images for each experiment were selected from those processed with three eyeballs.Ch, choroid; Ret, retina; CNV, choroidal neovascularization; RPE, retinal pigment epithelia; MMP-2, Matrix metallopeptidase-2. Scale bar = 50 μ.

Krypton laser photocoagulation was used to create CNV in the right eyes of six to eight week-old mice; the left eyes of the mice were used as controls. At 12 to 15 days after fundus photocoagulation, the cryosections from serial sections of the laser-treated eyes were examined with immunohistochemistry against CD31, an endothelial marker of blood vessels. The RPE layer was destroyed and CD31 staining was present in the subretinal space in the CNV group, indicating that laser treatment destroyed the choroid-RPE barrier at the laser spots and induced endothelial cells to migrate to the subretinal space, which is evidence that successful CNV was created (Figure 1 top row, right panel). To demonstrate the roles of MMP-2 in the process, the serial sections from the same sets of cryosections were subjected to MMP-2 staining; increased staining was detected in the choroid-RPE membrane (Figure 1 bottom row, right panel).

### Decreased miR-29s in choroid-RPE tissue of the CNV model

**Figure 2:**
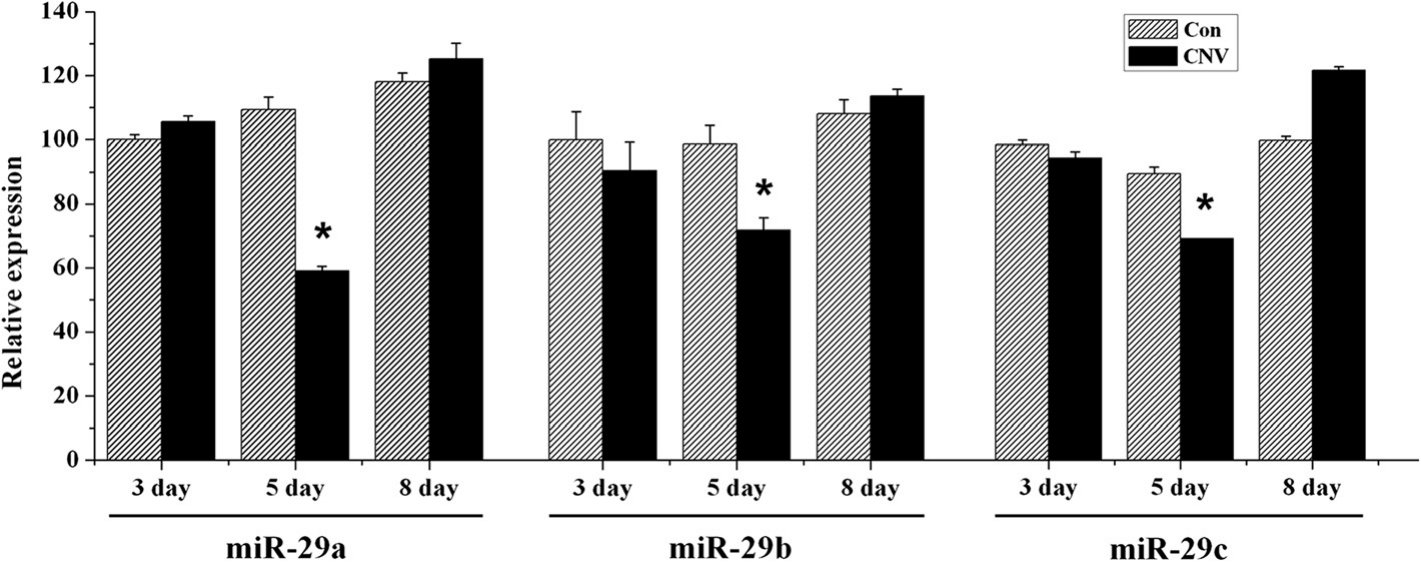
**Decreased miR-29 s level in choroidal-RPE tissue of CNV.** Choroidal-RPE tissues were isolated from non-treated and laser-treated eyeballs at days three, five and eight after photocoagulation and processed for miR-29s detection with RT-qPCR, as described in Methods. The values of control were set as 100% and the results from treatments were normalized to the control values. The results shown are mean (±SEM) averaged from three independent experiments with tissues for each experiment collected from between 5 and 7 eyeballs. *P < 0.05 versus control. CNV, choroidal neovascularization; RPE, retinal pigment epithelia.

Although miRNAs are involved in the complicated network of angiogenic regulation, the roles of miRNAs in the development of CNV are rarely characterized. Bioinformatic analyses with miRBase, Target Scan, and Pic Tar indicated that MMP-2 regulation could be mediated by miR-29s, and evidence indicated that miR-29b is involved in tumor angiogenesis, invasion, and metastasis by mediating MMP-2 protein expression [15]. Therefore, we decided to search for miR-29s in choroid-RPE tissue from the CNV model as a means of determining the possible roles of miR-29s in CNV. Since the amount of the choroidal-RPE tissue is far too low for northern blot detection, we used RT-qPCR to quantify the miR-29s. The linear amplification of RT-qPCR was tested with amplication of miR-29s, using various amounts of mouse cerebral tissues with weight differences in order (data not shown). Interestingly, we found that miR-29s including miR-29a, b, and c, at five days after photocoagulation, decreased significantly compared to the amounts in the contralateral eyes whereas at early (three days) or later time points (eight days), the miR-29s had not changed (Figure 2).

### NFkB activation in choroid-RPE tissue of the CNV model

**Figure 3:**
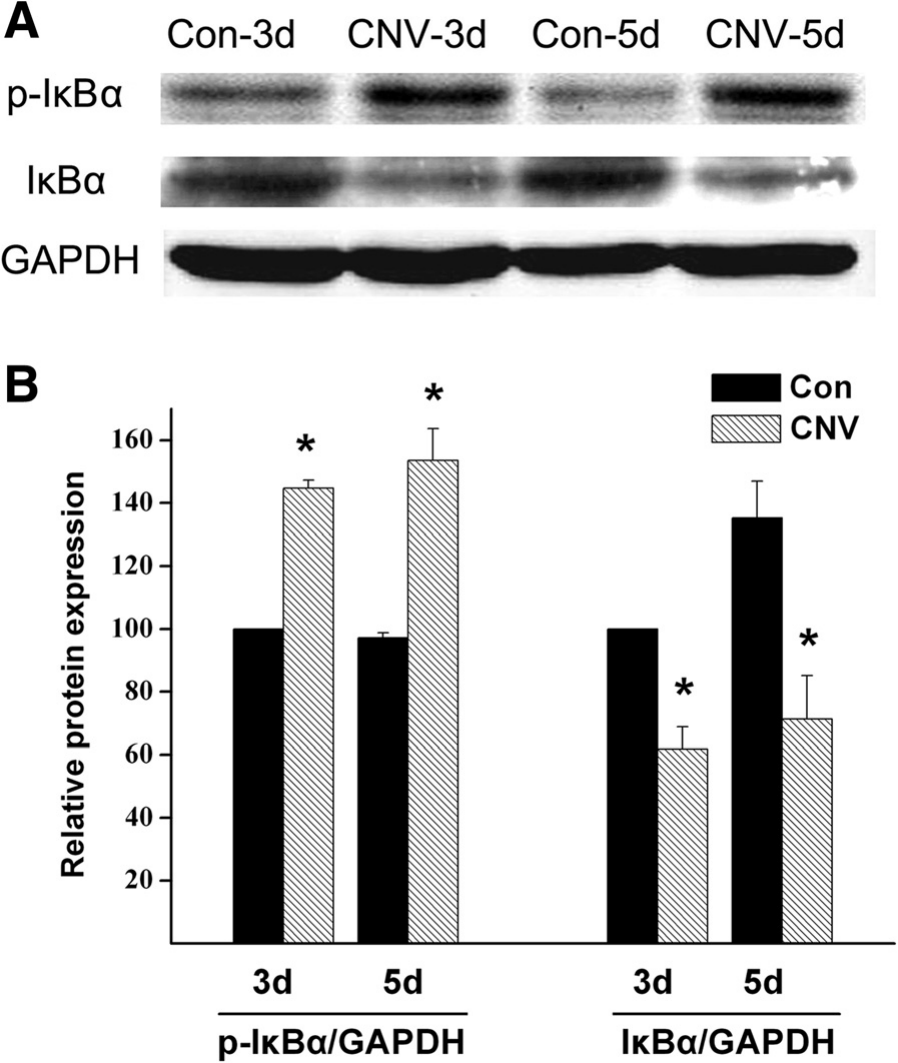
**Increased phospho-IκBα and decreased IκBα in choroidal-RPE tissues of CNV. A**, Choroidal-RPE tissues were isolated from non-treated and laser-treated eyeballs at days three and five after photocoagulation and processed for immunoblotting. The primary antibodies phospho-IκBα (1:200) and IκBα (1:200) and the peroxidase-conjugated secondary antibody (1:5000) were used, followed by ECL detection. The optical density ratios for p-IκBα/GAPDH and IκBα/GAPDH were indicated in **B**, the control values were set as 100 and the values for treated conditions were normalized to the control values. The images were typical of those from three independent experiments with tissues for each condition of per experiment collected from three eyeballs. *P < 0.05 versus the corresponding controls. CNV, choroidal neovascularization; RPE, retinal pigment epithelia; IκBα, nuclear factor of kappa light polypeptide gene enhancer in B-cells inhibitor, alpha; ECL, enhanced chemiluminescence assay; GAPDH, glyceraldehydes 3-phosphate dehydrogenase.

Recent studies suggested that the development of CNV is significantly suppressed by inhibiting the activation of NFκB [22,29,30]. NFκB complex is sequestered by inhibitors (IκB family proteins in cytoplasm in an inactive state) but phosphorylated by IκB kinase [31] under stimuli (phospho-IκB is subjected to degradation by proteasome complex). As a consequence, NFκB is released from IκB binding to enter the nucleus and activate transcription of specific genes. To determine whether NFκB was activated during the development of CNV, we analyzed phospho-IκBα and IκBα [32]. Western blot detected increased phospho-IκBα and decreased IκBα in choroid-RPE tissue of CNV model at days three and five after laser induction, compared with amounts in non-treated eyes, a finding suggesting that the laser treatment had activated the NFκB pathway in the choroid-RPE tissue (Figure 3).

**Figure 4:**
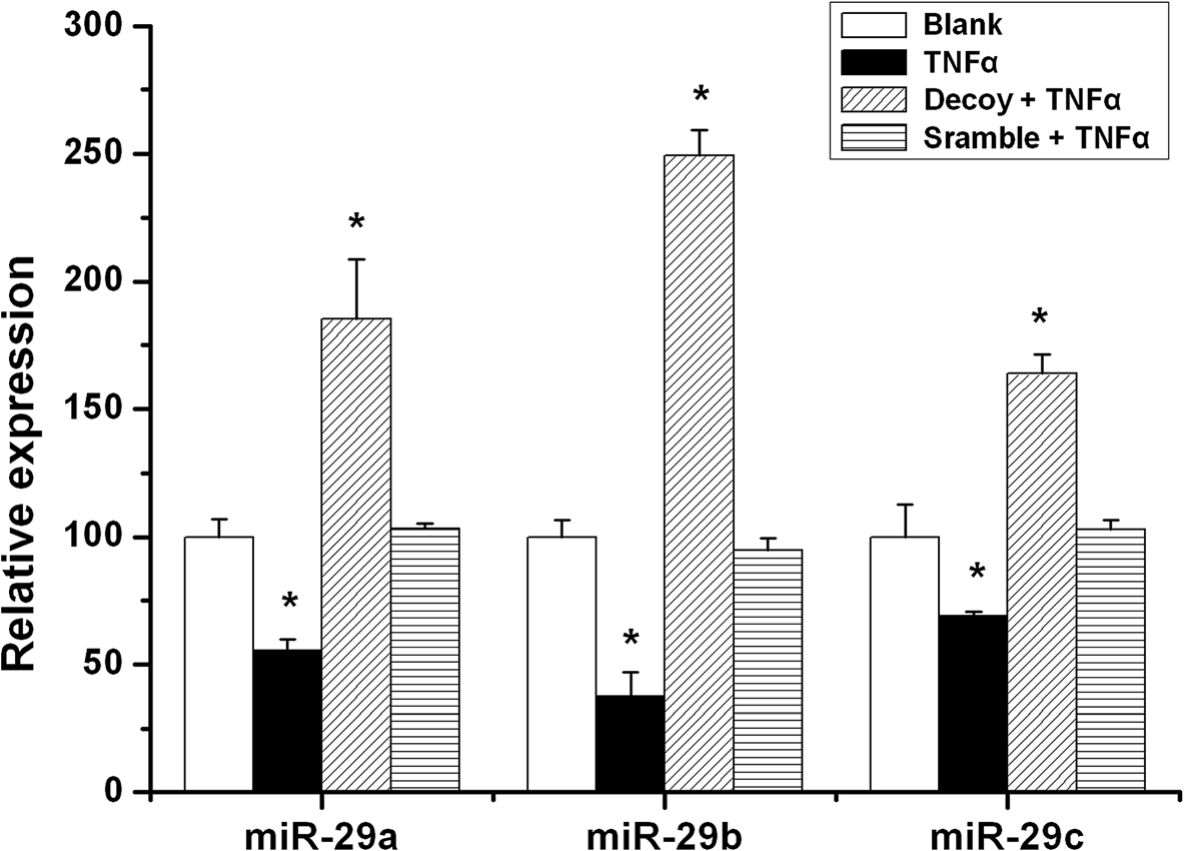
**TNFα reduced miR-29 s, which was reversed by NFκB decoy.** ARPE-19 cells were cultured in six-well dishes and transfected with NFκB decoy ODNs (50 nM) or scramble ODNs (50 nM) when the cell densities reached approximately 70% confluency. The ARPE-19 cells were then treated with TNFα (10 ng/mL) for 12 hours and miR-29 s levels were determined by RT-qPCR. The values of control were set as 100% and the results from treatments were normalized to control. The results shown are mean (±SEM) from at least three independent experiments with each condition per experiment from quadruplicate cultures. *P < 0.05 versus control. NFκB, nuclear factor of kappa light polypeptide gene enhancer in B-cells; ODN, oligodeoxynucleotide; TNFα, tumor necrosis factor alpha.

It was recently shown that the transcription factor NFκB negatively regulates miR-29 b/c in various cells [18,23]. We therefore examined the possible role of this pathway in the regulation of miR-29s in ARPE-19 cells, a human RPE cell line. Previous studies indicated that TNFα is significantly increased in choroidal-RPE tissue in laser-induced neovascularization mice model [33,34] and contributes to laser-induced CNV formation [35,36]. Consequently, we tested the possible regulatory effect of TNFα on the miR-29 family and determined stimulation with TNFα (10 ng/mL) resulted in significant down-regulation of all miR-29 members (Figure 4). Furthermore, transfection of the synthetic NFκB decoy, imitating the NFκB binding site that has been reported to inhibit NFκB activation [28], rescued the down-regulation of miR-29s by TNFα (Figure 4). These results were consistent with a previous study indicating that a chemical inhibitor of NFκB activation increases the expression of miR-29s [37].

### miR-29s down-regulated MMP-2 expression in RPE and endothelial cells

**Figure 5:**
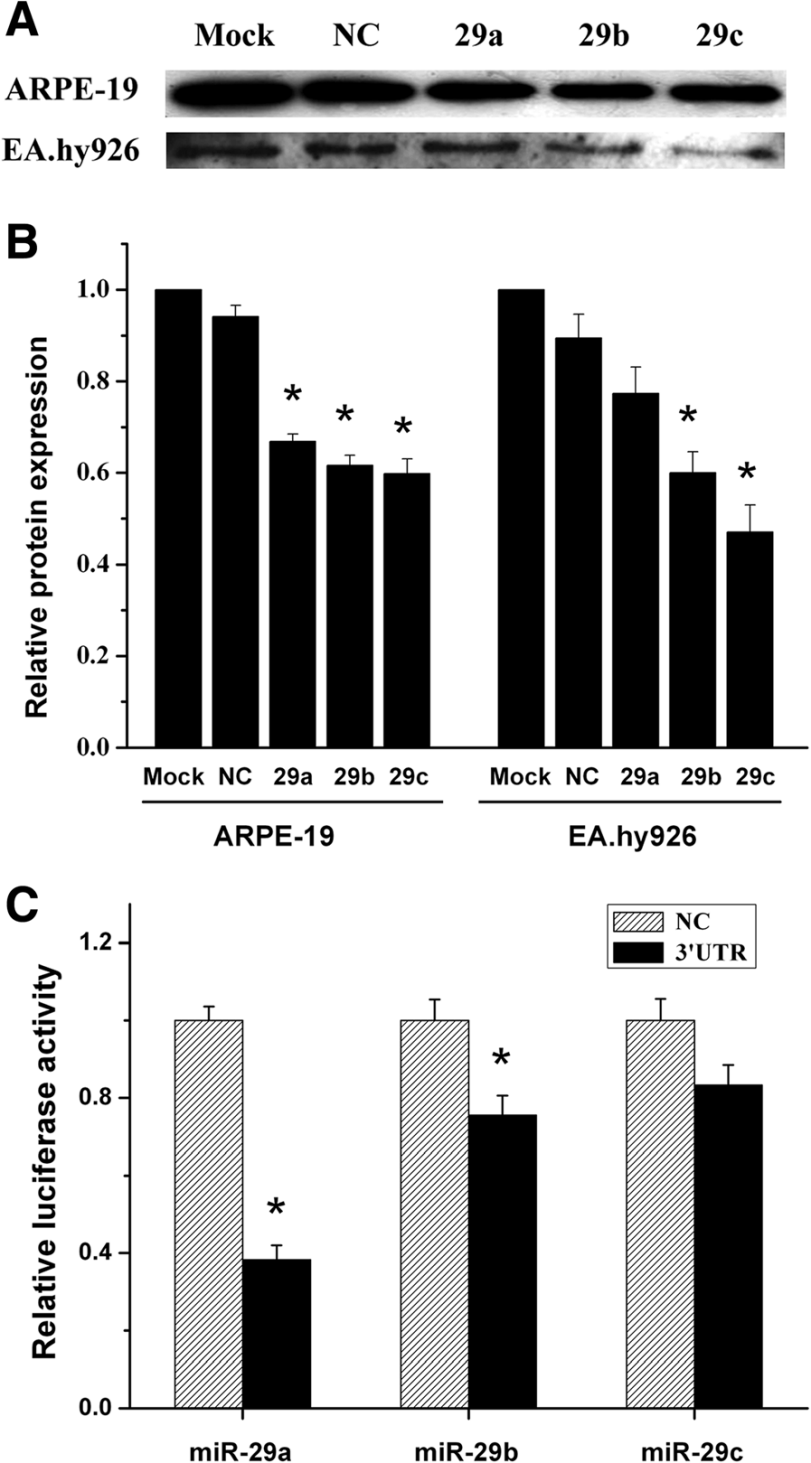
**miR**-**29 s down**-**regulated MMP-2 in ARPE-19 and EA hy926 cells. A**, ARPE-19 or EA hy926 cells were cultured in six-well dishes and transfected with miR-29a, b, and c (50 nM), and NC (50 nM) individually when the cell densities reached approximately 70% confluency. After being cultured for 48 hours, the supernatants were harvested for examining the MMP-2 contents with western blot as described in Methods. The images shown are typical of those from three independent experiments with each condition from triplicate cultures. **B**, The ratios of Mock between supernatant MMP-2 and protein mass in culture wells were set as 1 and the values for treated conditions were normalized to the control values. **C**, HEK-293 cells were cultured in 96-well dishes, and each well was transfected with 50 ng pMIR-MMP-2 3’UTR/firefly luciferase, 25 ng pRL-SV40 Renilla luciferase vector, and 5 nM miR-29 mimics or 5 nM NC. Luciferase activities were determined 24 hours after transfection and the results were expressed as the ratios between the activity of firefly luciferase and that of Renillaluciferase. The results shown were mean (±SEM) from three independent experiment with each condition per experiment from quadruplicate cultures. The values of control were set as 1 and the results from treatments were normalized to control. *P < 0.05 versus NC. NC, negative control; MMP-2, matrix metallopeptidase-2; UTR, untranslated region.

In choroidal-RPE tissue of the CNV model, the increased expression of the MMP-2 was accompanied with decreased expression of miR-29s (Figure 1 and Figure 2). Further bioinformatic analysis reveals a complementary sequence for all the miR-29 members in the 3’-UTR of MMP-2 (data not shown). We subsequently investigated the possibility that miR-29s down-regulate MMP-2 expression *in vitro*. Since RPE and endothelial cells are the major cell types secreting MMP-2 in choroid-RPE tissue, ARPE-19 and EAhy 926 were used to test whether the expression of MMP-2 was regulated by miR-29s. We transfected both types of cells with miR-29 mimics or non-specific control miRNA mimics (NC mimic). Transfection with individual miR-29 reduced protein levels of MMP-2 to a similar level, but transfection with miR-29 b/c induced a larger decrease in EA hy926 than did transfection with miR-29a (Figure 5A and B).

To confirm whether 3’-UTR of MMP-2 is a functional target of miR-29s, we cloned a reporter plasmid containing the 3’UTR of MMP-2 (pMIR-MMP-2 3’UTR, Methods) between the firefly luciferase reporter gene and polyA tail in pMIR-REPORT™. HEK-293 cells, which contain low levels of endogenous miR-29s, were transfected with individual miR-29a, b, and c mimics, pMIR-MMP-2 3’UTR, and Renilla luciferase reporter plasmids. The luciferase assay indicated that co-transfection of miR-29a and b mimics resulted in significantly decreased luciferase activity, whereas transfection of miR-29c mimic had a limited effect (Figure 5C).

## Discussion

Our results in the CNV model demonstrated that NFκB activation accompanied decreased miR-29s levels in choroidal-RPE tissue, which contained elevated levels of MMP-2 protein. At the cellular level, TNFα, an important cytokine in laser-induced CNV, decreased miR-29s in ARPE-19 cells. This effect was dependent on NFκB pathway activation. Finally, we confirmed that miRNA-29s down-regulated MMP-2 expression in the RPE and endothelial cells. Considering the critical roles of RPE and choroidal endothelial cells in angiogenic processes, our results suggested that NFκB-miR-29s-MMP-2 circuitry contributes to the angiogenesis in CNV.

Co-transfection with the NFκB decoy rescued the reduction of miR-29s by TNFα, and the effects exceeded the control level, possibly due to the complete blockade of NFκB pathway by the decoy (Figure 4). The miR-29s mimics a and b reduced secreted MMP-2 levels to the same extent as in ARPE-19 cells which is in contrast to the differential effects in EA hy926 and probably reflects the cell-type specificity of miRNA-29s (Figure 5). For instance, as reported, epigenetic modification by miR-29b is distinct from miR-29a and c [38], whereas miR-29a specifically modulates the angiogenic process in endothelial cells [39].

Although we demonstrated the possible roles of NFκB-miR-29s-MMP2 circuitry in CNV, the involved molecules and the associated regulatory mechanisms in CNV are versatile. For instance, NFκB pathway activation is associated with angiogenesis, and blockade of the NFκB pathway inhibits angiogenesis in various contexts [21,22]. One defined mechanism of MMP-2 regulation by NFκB activation is that the increasing transcription of MT1-MMP [40,41] cleaves pro-MMP-2 to its mature form, MMP-2 [42] (the pathway is independent of miR-29s). However, overproduced MMP2, either from direct activation of NFκB or the reduced level of miR-29s in various contexts, leads to enhanced degradation of the basement membrane and extracellular matrix, which are prerequisites for endothelial sprout invasion, an early event in angiogenesis [5]. In addition to the MMP family, including MMP-2 and MMP-9 [25] which are involved in CNV, the angiogenic process in CNV is complicated, involving a network of interactions between RPE cells and choroidal endothelial cells. These paracrine interactions were reportedly mediated by molecules secreted by either kind of cell, for example, the molecules related to the activation of the plasminogen/plasminogen system [40], angiogenic factor, VEGF [4,10], cytokine, TNFα [33], and monocyte chemotactic protein1 (MCP1) produced by invading macrophages at CNV sites [43].

It is clear that MMP-2 plays an essential role in regulating the angiogenesis in CNV, an opinion supported by the observations that CNV is less in MMP-2 deficient mice [8] and is decreased by anti-angiogenic approaches, including the use of MMP inhibitors targeting MMP-2, 9 and MT1-MMP [25]. Here, we demonstrated a possible MMP2 regulatory pathway *in vivo* and *ex vivo* in CNV via down-regulation of miR-29s mediated by NFκB activation. These results suggest a promising strategy for resolving CNV by targeting miR-29s levels.

## Abbreviations

AMD: Age-related macular degeneration; CNV: Choroidal neovascularization; miR-29: microRNA 29; HUVEC: Human umbilical vein endothelial cell; MMP-2: Matrix metallopeptidase-2; MT1-MMP: Membrane type1 metalloprotease; NFκB: Nuclear factor kappa-like-chain-enhancer of activated B cells; ODN: Oligodeoxynucleotide; RPE: Retinal pigment epithelial cell; RT-qPCR: Reverse transcription quantitative real-time PCR; TIMP: Tissue inhibitors of metalloproteinase; TNFα: Tumor necrosis factor alpha; VEGF: Vascular endothelial growth factor; UTR: untranslated region.

## Competing interests

The authors declare that they have no competing interests.

## Authors’ contributions

CJJ performed RT-qPCR, cloned MMP-2 luciferase assay reporter, did luciferase assay and western blot, analyzed the data, and contributed to the writing. YGB performed western blot, immunohistochemistry and miR-29s northern blot, and joined in the luciferase reporter cloning. LB created the CNV model. XW helped the operation during the procedure of laser photocoagulation. LXL provided CNV facilities. CXY and YDS provided technique support on miR-29s northern blot. Shan G and Qu J provided expert opinions on the projects. WS conceived of the project, designed the experiments, provided technique support, analyzed the data, and wrote the manuscript. All authors read and approved the final manuscript.
